# Spent Culture Medium from Virulent *Borrelia burgdorferi* Increases Permeability of Individually Perfused Microvessels of Rat Mesentery

**DOI:** 10.1371/journal.pone.0004101

**Published:** 2008-12-31

**Authors:** Xueping Zhou, Michael R. Miller, Md Motaleb, Nyles W. Charon, Pingnian He

**Affiliations:** 1 Department of Physiology and Pharmacology, West Virginia University, Morgantown, West Virginia, United States of America; 2 Department of Biochemistry, West Virginia University, Morgantown, West Virginia, United States of America; 3 Department of Microbiology, Immunology and Cell Biology, West Virginia University, Morgantown, West Virginia, United States of America; University of Liverpool, United Kingdom

## Abstract

**Background:**

Lyme disease is a common vector-borne disease caused by the spirochete *Borrelia burgdorferi* (*Bb*), which manifests as systemic and targeted tissue inflammation. Both *in vitro* and *in vivo* studies have shown that *Bb*-induced inflammation is primarily host-mediated, via cytokine or chemokine production that promotes leukocyte adhesion/migration. Whether *Bb* produces mediators that can directly alter the vascular permeability *in vivo* has not been investigated. The objective of the present study was to investigate if *Bb* produces a mediator(s) that can directly activate endothelial cells resulting in increases in permeability in intact microvessels in the absence of blood cells.

**Methodology/Principal Findings:**

The effects of cell-free, spent culture medium from virulent (B31-A3) and avirulent (B31-A) *B. burgdorferi* on microvessel permeability and endothelial calcium concentration, [Ca^2+^]_i_, were examined in individually perfused rat mesenteric venules. Microvessel permeability was determined by measuring hydraulic conductivity (Lp). Endothelial [Ca^2+^]_i_, a necessary signal initiating hyperpermeability, was measured in Fura-2 loaded microvessels. B31-A3 spent medium caused a rapid and transient increase in Lp and endothelial [Ca^2+^]_i_. Within 2–5 min, the mean peak Lp increased to 5.6±0.9 times the control, and endothelial [Ca^2+^]_i_ increased from 113±11 nM to a mean peak value of 324±35 nM. In contrast, neither endothelial [Ca^2+^]_i_ nor Lp was altered by B31-A spent medium.

**Conclusions/Significance:**

A mediator(s) produced by virulent *Bb* under culture conditions directly activates endothelial cells, resulting in increases in microvessel permeability. Most importantly, the production of this mediator is associated with *Bb* virulence and is likely produced by one or more of the 8 plasmid(s) missing from strain B31-A.

## Introduction

Lyme disease, caused by the spirochete *Borrelia burgdorferi (Bb)*, is the most common vector-borne disease in the US and Europe [1]. When infected *Ixodes* ticks feed on humans, the spirochetes move from the tick into the skin, and as the spirochetes move through the dermis, they often cause fever, skin rash (erythra migrans), and flu-like symptoms [2]. Within several days, *Bb* penetrates the vascular system and is disseminated to other tissues, inducing arthritis, cardiac complications, and neurological symptoms [1], [3]. The use of antibiotics usually reduces antibody titers, indicating a direct role of *Bb* in the manifestations of Lyme disease [3]. Fostered by environmental conditions, Lyme disease is predicted to be “a continuing public health concern” [3]. A better understanding of the mechanisms of *Bb*-induced inflammation and the factors that facilitate *Bb* penetration through vascular walls may benefit prevention of disease progression.

*Bb*-induced inflammation has been studied *in vitro*, by co-culturing *Bb* with endothelial monolayers and/or leukocytes, with a focus on proinflammatory cytokines, chemokines and other molecules produced by host cells that promote inflammation and leukocyte adhesion/migration [4]–[6]. Studies *in vivo* indicate the innate immune response plays a significant role in *Bb*-mediated inflammation (reviewed in [7], [8]). Some *Bb* lipoproteins interact with Toll-like receptors (TLR) in the innate immune response [9]–[12]. However, additional mechanisms may also contribute to *Bb*-mediated inflammation [12]–[14]. Few studies have examined the possibility that *Bb* may produce a mediator(s) that directly causes inflammation. *Bb* does not synthesize the classic LPS [15]–[18]. Although two *Bb* glycolipids have been identified, with some properties similar to LPS [18], it is not known if these glycolipids mediate inflammatory reactions. *In vitro*, a *Bb* lipoprotein has been shown to upregulate expression of endothelial cell E-selectin, VCAM-1, ICAM-1 and neutrophil migration [19]. Whether *Bb* lipoproteins, glycolipids or other mediators can directly alter the permeability of intact vascular walls has not been investigated. Studies conducted in individually perfused microvessels demonstrated that, in addition to inflammation mediated by leukocyte/endothelial cell interactions, endothelial cells can be directly activated by a variety of mediators, resulting in a rapid increase in microvessel permeability that is initiated by increases in endothelial [Ca^2+^]_i_ [20], [21]. If *Bb* produces a mediator(s) that can directly activate endothelial cells lining microvessel walls, it would not only cause leakage of excessive fluid and macromolecules, but the resulting barrier dysfunction may also facilitate *Bb* penetration and dissemination to targeted tissues. In addition, some of the 21 circular and linear plasmids (cp and lp, respectively) in *Bb* [16] are associated with infectivity, especially lp25, lp28-1, lp26 and cp26; presumably they encode virulence factors important in the pathogenesis of Lyme disease [22]–[25]. The extent to which *Bb* plasmids may be associated with *Bb*-induced inflammation *in vivo* remains unknown.

The objective of the present study was to investigate if *Bb* produces a mediator(s) that can directly increase permeability in intact microvessels. Cell-free, spent *Bb* culture medium was used to perfuse individually cannulated rat mesenteric venules. The changes in microvessel permeability were determined by measuring hydraulic conductivity (Lp) before and after each vessel was exposed to *Bb* spent medium, and associated changes in endothelial [Ca^2+^]_i_ were measured in Fura-2 loaded microvessels [26]. To investigate whether *Bb*-induced inflammation is associated with virulence, the effects of spent medium from both virulent (B31-A3) and avirulent (B31-A) strains on microvessel Lp and endothelial [Ca^2+^]_i_ were investigated. The plasmid content of strains B31-A3 and B31-A were determined by PCR analysis.

## Materials and Methods

### *Bb* strains and culture

*Bb* strains B31-A3 and B31-A were cultured in Barbour-Stonner-Kelly II medium containing 6% rabbit serum (BSK-II) at 33°C in a humidified chamber with 3% CO_2_. B31-A3, a low-passage, virulent, cloned strain derived from B3 MI that contains 20 circular and linear plasmids [22], was the generous gift from Dr. P. Rosa, Rocky Mountain Laboratories, and was propagated in culture for no more than 3 passages. B31-A is a cloned, high-passage, avirulent strain derived from B31 MI [27] that has lost multiple plasmids; the plasmid content of this strain is presented in this study. Cells were enumerated by flow cytometry [28]. B31-A3 and B31-A strains grew at similar rates, and spent medium was prepared when strains were at comparable cell densities.

### Spent-medium preparation

Both strains B31-A3 and B31-A were inoculated into BSK-II medium at low cell density and grown to late log/early plateau phase (∼1–3×10^8^ cells/ml); >95% of the cells were highly motile. Cells were removed and spent medium was prepared by centrifuging aliquots of cultures at ∼14,000×g for 8 min at room temperature and filtering supernatants through 0.1 µm filters into sterile plastic tubes. No cell growth was detected when filtered, spent medium was placed in an incubator for a month. The pH of spent medium was adjusted to 7.4–7.45, and the protein concentration of the medium was measured immediately before Lp measurements in individually perfused mesenteric venules, using a refractometer. BSK-II medium not inoculated with spirochetes but subject to the same incubation and filtering procedures as active cultures served as medium controls.

### PCR analysis of *Bb* plasmids

PCR was used to identify the plasmids retained in B31-A and B31-A3 strains. The PCR conditions were described previously [22], and used 50 µl of total reaction volume, GoTaq polymerase (Promega), and unique primer sets for the 21 different linear and circular plasmids; primer sequence information was generously provided by D. R. Akins, Oklahoma University, Oklahoma City, OK. PCR products were resolved by agarose gel electrophoresis and visualized by ethidium bromide staining.

### Animal Preparation

Experiments were carried out on Female Sprague-Dawley rats (2–3 mo old, 220 to 250 g, Hilltop Laboratory Animal, Scottdale, PA) anesthetized with pentobarbital sodium (65 mg/kg.body wt) given subcutaneously. Additional 3 mg doses were given as needed to maintain anesthesia. All procedures and animals used were approved by the Animal Care and Use Committee at West Virginia University, in compliance with all relevant federal guidelines and institutional policies. Each rat was anesthetized, then the trachea was intubated, and a midline surgical incision (1.5 to 2 cm) was made in the abdominal wall. The mesentery was gently taken out from the abdominal cavity and spread over a pillar for measurements of Lp or over a glass coverslip attached to an animal tray for measuring endothelial [Ca^2+^]_i_. The upper surface of the mesentery was continuously superfused with mammalian Ringer solution at 37°C. All experiments were carried out in venular microvessels with diameters ranging between 35 and 50 µm, with one experiment per microvessel in each animal.

### Measurement of hydraulic conductivity (Lp)

**Figure 1:**
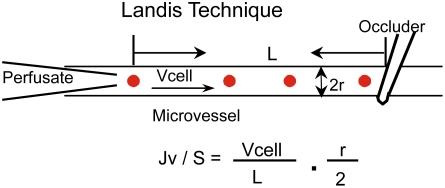
Hydraulic conductivity, Lp, was measured based on the modified Landis technique in individually perfused microvessels. Jv is the initial water flow per unit area of microvessel wall (S), which was calculated from the velocity of the marker cell (Vcell) after the vessel was occluded, the vessel radius (r), and the length between the marker cell position and the occlusion site (L).

All measurements were based on the modified Landis technique that measures water flux across the microvessel wall. The assumptions and limitations of the original method and its application in mammalian microvessels have been evaluated elsewhere [29]. Briefly, a single venular microvessel was cannulated with a glass micropipette filled with albumin-Ringer solution (control) containing 1% (v/v) hamster red blood cells as markers. The micropipette was connected to a water manometer. A hydrostatic pressure (range 30–70 cmH_2_O) was applied through the micropipette to the microvessel lumen to allow continuous flow of perfusate in the vessel. A CCD camera was connected to the microscope, and video images were continuously recorded from a segment of the perfused microvessel throughout each experiment for data analysis. For each measurement, the perfused vessel was occluded downstream with a glass rod for 5–7 sec and then the rod was released to allow perfusate flow for at least 15 sec before another occlusion was made. The initial water flow per unit area of microvessel wall [(Jv/S)_0_, where Jv is water flux and S is unit area of the microvessel wall] was calculated from the velocity of the marker cell after the vessel was occluded, the vessel radius, and the length between the marker cell position and the occlusion site (Fig. 1). Microvessel Lp was calculated from the Starling equation, Lp = (Jv/S)_0_, /ΔP, where ΔP is the pressure difference between the hydrostatic pressure applied to the microvessel and the effective oncotic pressure generated from protein in the perfusate. The effective oncotic pressure with bovine serum albumin at 10 mg/ml is 3.6 cmH_2_O and at 50 mg/ml is 21 cmH_2_O [30]. The oncotic pressures in BSK II medium and *B. burgdorferi* spent medium were estimated accordingly, based on their measured protein concentrations ranging from 45–55 mg/ml.

### Measurements of endothelial [Ca2+]i

**Figure 2:**
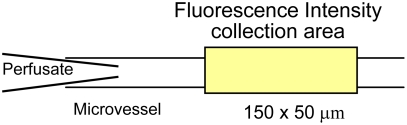
Measurements of changes in endothelial [Ca^2+^]_i_ in an individually perfused microvessel. The vessel was first perfused with albumin-Ringer solution containing Fura 2-AM for 45 min to load the endothelial cells of the microvessel wall. The vessel was then recannulated and perfused with albumin-Ringer solution for 10 min to remove fura 2-AM from the vessel lumen. Fluorescence intensity was collected through a rectangular diaphragm of the photometer (yellow area) under control conditions and after exposure to testing solutions.

Endothelial cell [Ca^2+^]_i_ was measured in individually perfused microvessels using the fluorescent Ca^2+^ indicator fura 2-AM, as described [20], [26]. Experiments were performed on a Nikon Diaphod 300 microscope equipped with a Nikon photometry system, computer-controlled shutter, and filter changer (Lambda 10-2; Sutter Instrument; Novato, CA). Briefly, a venular microvessel in rat mesentery was cannulated and perfused first with albumin-Ringer solution containing 10 µM fura 2-AM for 45 min. The vessel was then recannulated and perfused with albumin-Ringer solution for 10 min to remove fura 2-AM from the lumen. Fluorescence intensity was collected by a Nikon Fluor lens (×20, NA 0.75) through a rectangular diaphragm of the photometer (∼150×50 µm) (Fig. 2). The excitation wavelengths for fura 2-AM were selected by two narrow-band interference filters (340±5 and 380±5 nm; Oriel) from a Xenon (75 W) light source, and the emission was separated with a dichroic mirror (DM400) and a wide-band interference filter (500±35 nm; Oriel). The excitation wavelength alternated between 340 and 380 nm, and corresponding FI values (FI_340_ and FI_380_, respectively) were collected with a 0.25-s exposure at each wavelength. At the end of each experiment, the microvessel was superfused with a modified Ringer solution (5 mM Mn^2+^ without Ca^2+^) while being perfused with the same solution containing ionomycin (10 µM) to bleach the Ca^2+^-sensitive form of fura 2. The background FIs due to unconverted fura 2-AM and other Ca^2+^-insensitive forms of fura 2 were subtracted from FI_340_ and FI_380_ values. The ratios of the two FI values were converted to Ca^2+^ concentrations using an *in vitro* calibration curve [20].

### Data analysis and statistics

PCR analysis of plasmid contents for B31-A3 and B31-A strains were repeated three times to assure reproducibility. The Lp changes of each vessel after exposure to testing solutions were expressed as Lp_test_/Lp_control_. Values are expressed as means±SE. If Lp is relatively constant throughout the time course, the mean Lp value for each perfusate was calculated from all of the occlusions during that perfusion period. If a transient increase in Lp is observed, Lp is reported as the means of peak values. For statistical comparisons, a paired t-test was used for the mean values obtained before and after stimulation from the same vessel. An unpaired t-test and one way ANOVA were used to compare mean values among groups. A probability value of P<0.05 was considered statistically significant. In summary figures, * indicates a significant increase from the negative control, and † indicates significantly lower than the positive control.

## Results

### Plasmid profiles of B31-A3 and B31-A

**Figure 3:**
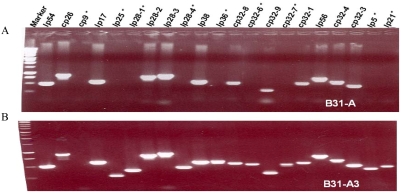
PCR analysis of plasmids in strains B31-A and B31-A3. Twenty-one pairs of specific primers were used to amplify all the circular and linear plasmids contained in the two strains. DNA markers (left lane) identify the sizes of amplified DNAs. “*” indicates the plasmids that are lost from B31-A strain. A) B31-A is missing lp25, lp28-1, lp28-4, lp36, cp32-6, cp32-7, lp5 and lp21, in addition to cp9. B) B31-A3 retains all twenty one plasmids except cp9, as expected [22].

PCR analysis demonstrated that our B31-A strain has lost eight plasmids (lp5, lp21, lp25, lp28-1, lp28-4, cp32-6, cp32-7 and lp36) retained in B31-A3 and confirms that strain B31-A3 retains all plasmids associated with the original virulent B31 MI isolate except cp9 [22] (Fig. 3). Thus, a major difference between these two strains is their plasmid content.

### B31-A3 spent medium increases permeability in individually perfused intact venules

**Figure 4:**
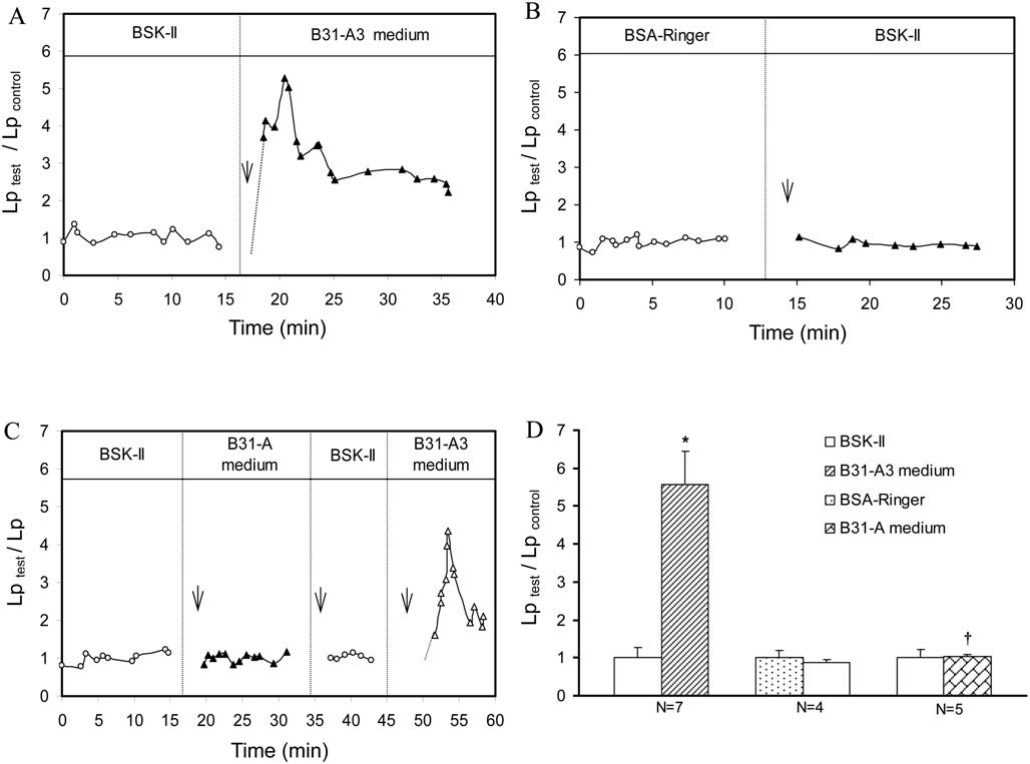
Effects of B31-A3, B31-A spent medium and BSK-II control medium on Lp. Relative changes in Lp are presented as Lp_test_/Lp_control_. Arrows indicate times at which perfusion with test solutions were initiated. A) One representative experiment showing a transient increase in Lp induced by B31-A3 spent medium. B) A representative experiment showing Lp is unaffected when vessels were perfused with BSK-II medium. C) Time course and magnitude changes in Lp from one representative paired experiment in which a venule was sequentially perfused with BSK-II medium, B31-A spent medium, BSK-II medium, then B31-A3 spent medium. An increase in Lp was only seen upon perfusion with B31-A3 spent medium. D) Summarized data of Lp changes induced by B31-A and B31-A3 spent medium; * indicates a significant increase (P<0.05) from negative control; † indicates a significant decrease (P<0.05) from B31-A3 spent medium.

The effect of spent medium from cultured B31-A3 cells on microvessel permeability was investigated in seven individual venular microvessels. Fig. 4A shows a representative magnitude and time course of Lp response to B31-A3 spent medium from one of the vessels. The mean control Lp, measured with BSK-II culture medium, was 1.1±0.3×10^−7^ cm.s^−1^.cmH_2_O^−1^. Exposing each vessel to B31-A3 spent medium caused a transient increase in Lp. The mean peak Lp, occurring 2–5 min after the start of B31-A3 spent medium perfusion, was 5.6±0.9 times that of the medium control (Fig. 4D, N = 7)

The effect of BSK-II medium on microvessel Lp was examined in 4 additional venular microvessels. Fig. 4B shows the Lp measurements from a single experiment. The mean control Lp of 4 vessels measured with albumin-Ringer perfusate was 1.8±0.3×10^−7^ cm.sec^−1^.cmH_2_O^−1^. After each vessel was recannulated and perfused with BSK-II medium, the mean Lp was 1.5±0.2×10^−7^ cm.sec^−1^.cmH_2_O^−1^, which was not significantly different from the Lp control level (Fig. 4D, N = 4, P>0.05), indicating that components in BSK-II medium had no effect on basal Lp.

### B31-A spent medium does not alter microvessel permeability

The effect of B31-A spent medium on micovessel permeability was investigated in 5 microvessels. The mean control Lp with BSK-II medium perfusion was 2.2±0.6×10^−7^ cm.s^−1^.cmH_2_O^−1^. When each vessel was recannulated and perfused with B31-A spent medium, Lp did not change (summarized in Fig. 4D, N = 5, P>0.05). In 3 of the 5 vessels, B31-A and B31-A3 spent medium were applied to the same vessel sequentially. Perfusion of B31-A spent medium did not increase Lp. Each vessel was then rinsed with BSK-II medium and exposed to B31-A3 spent medium. A significant increase in Lp was observed in each vessel, and the mean peak Lp was 4.6±0.5 times that of control. Fig. 4C shows a direct comparison of the effect of B31-A and B31-A3 spent medium on Lp in the same vessel.

### B31-A3 spent medium induces a transient increase in endothelial [Ca^2+^]_i_

**Figure 5:**
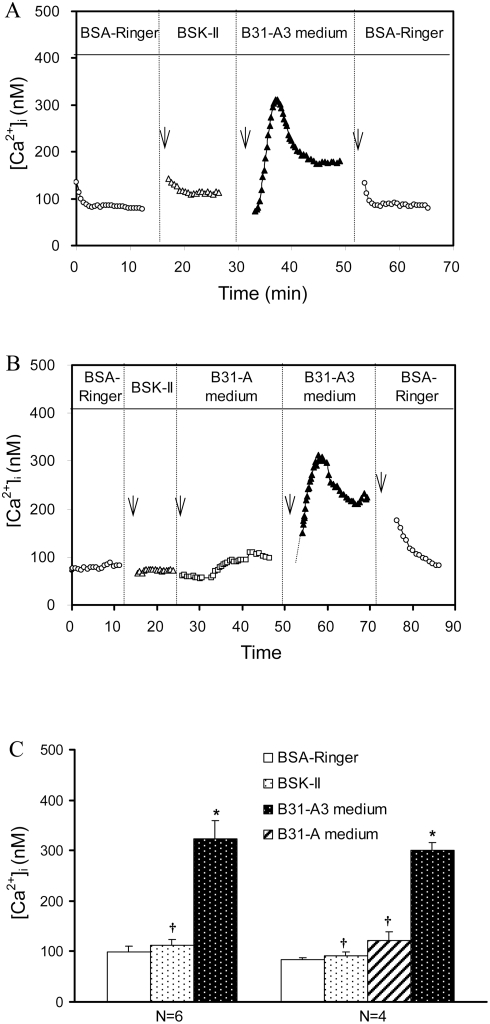
Effects of *Bb* spent medium on endothelial [Ca^2+^]_i_. A) A representative venule was sequentially perfused with 5% albumin-Ringer (5% BSA Ringer), BSK-II medium, B31-A3 spent medium and 5% albumin-Ringer, showing no endothelial [Ca^2+^]_i_ changes upon exposure to BSK-II medium, while a transient increase in endothelial [Ca^2+^]_i_ was observed within 2–3 min after exposure to B31-A3 spent medium. B) Changes in endothelial [Ca^2+^]_i_ from one representative paired experiment, showing no significant changes in endothelial [Ca^2+^]_i_ during perfusion of B31-A spent medium, while a transient increase in endothelial [Ca^2+^]_i_ occurred upon perfusion of B31-A3 spent medium. Arrows indicate times at which perfusion with test solutions were initiated. C) Summarized data for the effects of spent medium from B31-A3 and B31-A on endothelial [Ca^2+^]_i_; * indicates a significant increase (P<0.05) from negative control; † indicates a significant decrease (P<0.05) from B31-A3 spent medium.

The effects of B31-A3 spent medium on endothelial [Ca^2+^]_i_ were studied in 6 venules. A representative time course and magnitude changes in endothelial [Ca^2+^]_i_ is shown in Fig. 5A. The mean baseline [Ca^2+^]_i_ measured with albumin-Ringer perfusate was 99±12 nM (Fig. 5C, N = 6). Each vessel was then recannulated and perfused with BSK-II medium followed by perfusion with B31-A3 spent medium. The mean endothelial [Ca^2+^]_i_, measured with BSK-II medium was 113±11 nM, which was not statistically different from the albumin-Ringer control (P>0.05). A significant increase of endothelial [Ca^2+^]_i_ was observed when each vessel was perfused with B31-A3 spent medium (Fig. 5A and Fig. 5C, N = 6). Endothelial [Ca^2+^]_i_ reached a mean peak value of 324±35 nM in the first 2–3 minutes of B31-A3 spent medium perfusion, and fell to a sustained level (160±22 nM) after 10 min. When each vessel was recannulated and perfused with albumin-Ringer solution, [Ca^2+^]_i_ returned to the baseline level, 86±4 nM (Fig. 5A).

### B31-A spent medium does not alter endothelial [Ca^2+^]_i_

The effects of B31-A and B31-A3 spent medium on endothelial [Ca^2+^]_i_ were directly compared in four venules. A representative change in magnitude and time course of endothelial [Ca^2+^]_i_ is shown in Fig. 5B. The mean baseline [Ca^2+^]_i_ measured with albumin-Ringer solution was 83±4 nM. When each vessel was recannulated and perfused with BSK-II medium the mean [Ca^2+^]_i_ was 91±8 nM, which was not significantly different from the baseline (Figs. 5B and 5C, N = 4, P>0.05). Perfusion of each vessel with B31-A spent medium also did not induce a significant increase in endothelial [Ca^2+^]_i_, with a mean value of 127±17 nM (Fig. 5C, N = 4, P>0.05). Each vessel was then perfused with B31-A3 spent medium; the measured endothelial [Ca^2+^]_i_ reached a mean peak value of 301±15 nM within 3 min of B31-A3 spent medium perfusion (Fig. 5B and Fig. 5C, N = 4). These results show that B31-A3 spent medium increased endothelial [Ca^2+^]_i_, while spent medium from B31-A did not have an effect.

## Discussion

Our studies demonstrate for the first time that cell-free, spent medium from virulent *Bb* induces a rapid and transient increase in endothelial [Ca^2+^]_i_ and microvessel permeability (Lp) in rat mesenteric venules in the absence of blood cells. The time course and magnitude of increased Lp and [Ca^2+^]_i_ produced by *Bb* spent medium are similar to those induced by direct-acting inflammatory mediators, such as bradykinin or platelet activating factor (PAF) [20], [21]. This rapid and transient endothelial response to *Bb* spent medium in intact microvessels is distinct from other reports of *Bb*-mediated activation of cultured endothelial monolayers that usually occurs hrs after incubation with *Bb* [4], [5], [19], [31], [32]. Because the permeability effect of the spent medium was observed with virulent *Bb* but not avirulent *Bb*, the production of this mediator under culture conditions may be associated with *Bb* virulence, and appears to be produced by one or more of the 8 plasmids missing from our B31-A strain. We note that different plasmid contents have been reported for strain B31-A. This report and others [33], [34] finds B31-A is missing lp21, lp25, lp28-1, lp28-4, lp-36, cp9 and cp32-6. However, Lawrenz et al [33] report their B31-A strain is also missing lp28-3, while our strain is also missing lp5 and cp32-7 (apparently the other studies [33], [34] did not examine the presence of lp5). Thus B31-A is not a stable strain and it's plasmid content should be verified.

Previous studies demonstrated that the addition of *Bb* or a *Bb*-lipoprotein to cultured endothelial monolayers induces endothelial cells to produce proinflammatory cytokines, chemokines, adhesion molecules and chemotaxis activity, promoting transendothelial migration of lymphocytes and neutrophils [4], [5], [19], [31], [32]. Such inflammatory reactions are dependent on endothelial or host cells to produce inflammatory mediators and are only manifest after hrs. Although incubating cultured endothelial cell monolayers with *Bb* induced alterations of the endothelial barrier function, measured by electric cell substrate impedance sensing, the barrier function was not altered until five hr after addition of spirochetes [6]. In contrast, the increases in microvessel Lp and [Ca^2+^]_i_ upon addition of *Bb* spent medium peaked 2–5 min after the exposure (Figs 4 and 5), and thus appear to be a direct effect of the *Bb*-produced mediator under culture conditions.

Because the microvessel permeability effect was detected in cell-free spent medium, this mediator may be secreted; alternatively, it may be associated with membrane blebs that could pass through 0.1 µm filters [35], or be released by lysis of some cells during *in vitro* growth, or due to consumption of a factor in BSK-II medium by B31-A3 but not B31-A cells. While many pathogenic organisms produce exotoxins, endotoxins or other virulence factors that may directly induce inflammation, *Bb* is generally assumed not to produce toxins or directly cause tissue damage [16], [17], [36]. Analysis of the *Bb* genome indicated no classic toxins or virulence factors were encoded on the chromosome or 21 plasmids that would directly mediate inflammation [16], [17]. Our results also show that the transient increases in endothelial [Ca^2+^]_i_ and Lp induced by *Bb* spent medium are distinct from the irreversible increases in permeability induced by epsilon toxin, the direct-acting, primary virulence factor for *Clostridium perfringe* [37]. Only 2–6% of the 670 potentially intact genes on all 21 plasmids were similar to sequenced genes from other species with known and unknown functions [16]. Therefore, the microvessel-inflammatory mediator produced by *Bb* may be a novel type of inflammatory compound. More importantly, this mediator may be associated with *Bb* virulence and may be produced by one or more of the 8 plasmid(s) missing from strain B31-A. However, it should be noted that a chromosomal or plasmid mutation, independent of the 8 missing plasmids, may also contribute to the lack of mediator production by strain B31-A. Crude or undefined preparations, such as spent medium used in this study, are often the initial source for detecting a novel biological activity. Combined biochemical and genetic approaches will be applied in future studies to identify and characterize the source and chemical nature of this *Bb*-produced permeability-increasing mediator.

The type of inflammation observed in our studies with *Bb* spent medium could be difficult to detect in other systems, including whole animal or cell culture systems. The individually perfused microvessels have some unique advantages over other methodologies to detect the inflammatory mediator produced by *Bb*. First, the effect of the spent medium was examined in intact microvessels that have normal baseline permeability properties when experiments are initiated. Second, the single vessel perfusion technique allows microvessel permeability to be measured at a known perfusion pressure and the surface area for water and solute exchange can be measured precisely, which enables distinguishing changes in microvessel permeability from variations of flow dynamics and surface area under inflammatory conditions. Third, the changes in permeability with *Bb* spent medium were compared with its own control in the same vessel, which minimized the variability between vessels and animals. Most importantly, *Bb* has been reported to play significant roles in leukocyte adhesion and migration initiated by host-derived proinflammatory cytokines or chemokines [6], [38]. Our experiments, conducted in the absence of blood cells, enable the study of the direct effect of *Bb-*produced mediator on the endothelium independent of any permeability changes caused by leukocyte/endothelium interactions. The immediate, transient increases in Lp observed with B31-A3 spent medium perfusion indicate that *Bb* not only can induce the expression of adhesion and proinflammatory molecules in endothelia resulting in leukocyte adhesion and migration [6], [38], but may directly induce increases in microvessel permeability through calcium-associated endothelial cell activation.

Previous studies demonstrated that inflammatory mediators such as PAF, bradykinin, ATP, serotonin, and histamine, induce transient increases in microvessel permeability [39]–[41], and most of the permeability increases were initiated by increased endothelial [Ca^2+^]_i_ via increased Ca^2+^ influx [20], [21], [26], [42]. Our recent study further demonstrated that PAF-induced permeability increases are the result of gap formation between endothelial cells, which is critical for the subsequent formation and adhesion of leukocyte/platelet aggregates [43], [44]. The rapid induction of microvessel permeability induced by *Bb* spent medium, and the similarity in pattern to increased Lp and endothelial [Ca^2+^]_i_ induced by direct-acting inflammatory compounds [20], [21] suggests the permeability increases caused by the *Bb*-produced mediator may also be the result of endothelial gap formation. Our culture conditions utilized much higher concentrations of *Bb* than are encountered under physiological conditions. In Lyme disease (lower *Bb* densities), changes in microvessel permeability may be more subtle and may directly associate with the local adhesion and penetration of *Bb* across microvessel walls. Recent studies by Moriarty et. al. [45] and Norman et. al [46] demonstrated an *in vivo* approach to visualize spirochete interactions with endothelial cells and reported a fibronectin and sulfate-type glycosaminoglycan-dependent adhesion process. Combining *in vivo* approaches with fluorescence microscopy may allow further elucidation of subtle and local changes in endothelial junctions and permeability that may be associated with *Bb* adhesion and penetration processes. Weakening endothelial cell-cell junctions may play an essential role in *Bb* penetration into the vascular system for dissemination or possibly to escape from the vasculature to colonize susceptible tissues, including joints, the nervous system and heart.
